# US and Canadian cat caregiver’s ratings of cat-cat interactions: A video-based survey

**DOI:** 10.1017/awf.2024.58

**Published:** 2024-12-20

**Authors:** Sherry Khoddami, Makayla C. Kiser, Carly M. Moody

**Affiliations:** 1 Faculty of Land and Food Systems, University of British Columbia, Vancouver, BC, Canada; 2 Department of Animal Science, University of California, Davis, CA, USA

**Keywords:** affiliative behaviours, agonistic behaviours, animal welfare, cat behaviour, cat owner, cats

## Abstract

US and Canadian caregivers (n = 6,529) of two domestic cats (*Felis catus*) were recruited to participate in an online cross-sectional questionnaire to assess: (1) knowledge of inter-cat behaviour; (2) the frequency of positive and negative cat-cat interactions in the home; and (3) factors associated with positive and negative cat-cat interactions in the home. The questionnaire included ten videos (five negatively valenced, five positively valenced), in which participants scored: the overall cat-cat interaction; cat 1’s experience; and cat 2’s experience, using a Likert scale. Participants were also asked to report how often they see each interaction in their own two cats. Cat behaviour experts (n = 5) were recruited to rate their interpretations of the videos using the same Likert scale as the cat caregiver participants. Overall, our results suggest that overt positive interactions (allo-grooming, co-sleeping) were more likely reported if cat dyads were related or spent more time living together, were neutered males, indoor-only, and/or had a single feeding area. Overt negative interactions (fighting, striking) were more likely reported if dyads were older or had a larger age gap, showed animal-directed aggression, were declawed, and/or had a single litter-box. Participant versus expert ratings of the videos were similar, however caregivers reported certain affiliative behaviours more positively than experts. Caregivers appeared to have a good understanding of their cats’ overall relationship, as this aligned with reported cat-cat interactions. These results increase our understanding of the cat-cat relationship in two-cat households, which may be used to inform cat adoption strategies, in-home management, and promote a positive cat-cat relationship.

## Introduction

In the domestic cat (*Felis catus*), normal social behaviour includes a variety of affiliative and agonistic behaviours with other group members (Crowell-Davis *et al.* 2004). Affiliative behaviours are essential for maintaining social bonds (Bradshaw 2016; Vitale 2022) including allo-grooming, allo-rubbing, sleeping with contact, nose touching, and social play (Crowell-Davis *et al.* 2004; Overall 2013). Agonistic displays include behaviours such as resource guarding, staring, avoidance, displacement, striking, and negative vocalisations (Overall 2013). Certain interactions may also fall in-between affiliative and agonistic. Recently, an intermediate category has been suggested to exist between mutual social play and agonism, described by an extended period of inactivity, vocalisation, and chasing (Gajdoš-Kmecová *et al.* 2023). Evidently, the social behavioural repertoire of cats is complex, and the valence of certain interactions may be ambiguous or subtle, especially to an untrained observer. It is important that caregivers are able to detect and understand both subtle and obvious cat-cat interactions in the home, to manage the cat relationship and intervene before negative interactions develop into health and behaviour problems (Jones *et al.* 1997; Pryor *et al.* 2001; Barcelos *et al.* 2018). Furthermore, cat-cat interactions vary day-to-day and are subject to changes over time. Thus, for a holistic assessment of cat-cat relationships, behaviourists suggest caregivers should consider several interactions between their cats over time and monitor their relationship for signs of conflict (Crowell-Davis & Stelow 2023). For instance, cats with a positive relationship regularly engage in affiliative behaviours and may occasionally engage in agonistic displays, although less frequently (Elzerman *et al.* 2020; Crowell-Davis & Stelow 2023). Conversely, cats that are not getting along, frequently display signs of conflict or tension, including: maintaining distance from each other, avoiding close physical contact (such as allo-rubbing and co-sleeping), and avoiding playful interactions (Crowell-Davis & Stelow 2023; Gajdoš-Kmecová *et al.* 2023).

The inter-cat relationship may be influenced by several factors, many of which have not been explored in the home setting. For instance, behavioural observations of a neutered cat colony by Curtis and colleagues (2003) suggests cats that are related or familiar were more likely to keep a close distance and engage in affiliative interactions than unrelated cats. In large cohorts of indoor neutered cats, access to a larger space (a minimum of 4 m^2^ per cat) is shown to increase affiliative behaviours, such as play, allo-grooming, and body-contact compared to cats with less space (Loberg & Lundmark 2016). In addition to providing adequate space for cats to avoid inter-cat conflict and choose their affiliates, it is important to provide a sufficient quantity of dispersed resources such as litter-boxes, food and water bowls, scratchers, beds, hides, and perches. In neutered cat colonies, certain individuals monopolise resources, and thus multiple resources are recommended for equal access (Damasceno & Genaro 2014). In addition, cats may prefer to time-share resources, occupying them at different times (Bernstein & Strack 1996). Therefore, behaviourists commonly recommend providing multiple (i.e. quantity of litter-boxes should equal the number of cats in the home plus one extra), well-dispersed resources to help prevent and manage inter-cat conflict (Crowell-Davis & Stelow 2023). Despite this recommendation, many multi-cat households provide a single litter-box and feeding area (Alho *et al.* 2016; Grigg & Kogan 2019; Elzerman *et al.* 2020; Lawson *et al.* 2020; Khoddami *et al.* 2023), suggesting that cat caregivers may lack knowledge of their cats’ behavioural needs.

Research suggests that cat caregivers are more likely to view their cat’s behaviour as problematic if they display inter-cat aggression (Powell *et al.* 2023); a common issue faced by caregivers of multiple cats (Amat *et al.* 2009; Wassink-van der Schot *et al.* 2016; Roberts *et al.* 2020). It is important for cat caregivers to identify subtle signs of inter-cat conflict before it escalates to overt aggression. Excessive agonistic interactions that are not addressed may develop into illness, such as feline idiopathic cystitis, and behaviour problems such as house soiling (Jones *et al.* 1997; Pryor *et al.* 2001; Barcelos *et al.* 2018) which has been associated with an increased risk of relinquishment (Salman *et al.* 2000). Despite the importance of identifying conflict early on, few studies have explored cat caregivers’ knowledge of cat behaviours in the household. One study, conducted in Italy, suggests that cat caregivers may not identify the following negative cat responses as possible signs of stress: hiding (45.4%), dilated pupils (35.6%), and freezing (43.8%; Mariti *et al.* 2017). Other research by Dawson and colleagues (2019) conducted a survey to examine people’s abilities to identify the valence of cat facial expressions from videos, and the results showed that average scores were above chance, albeit low (11.85/20 correct). Furthermore, being a cat caregiver had little effect on participant scores, while professional cat-related experience (i.e. veterinary) did positively impact scores (Dawson *et al.* 2019). In addition, Van Belle and colleagues (2023) compared cat caregiver reports with video observations of their cats’ affiliative behaviours and found caregivers often under-report head rubbing and allo-grooming. This suggests that caregivers may not be able to identify positively valenced cat behaviours involved in affiliative interactions. To date, little research has focused on cat-cat interactions of one group size nor explored the ability of cat caregivers to interpret both negative and positive cat-cat interactions.

The current research focused on two-cat households in the US and Canada, given that many cat households in these countries contain two cats (Canadian Federation of Humane Societies [CFHS] 2017; Larkin 2021). In addition, it is well known that the size of a social group is known to impact social interactions and social complexity (Kappeler 2019). Given the limited research examining cat caregivers’ interpretations of inter-cat behaviour, we surveyed US and Canadian caregivers of two cats to: (1) assess knowledge of inter-cat behaviours through ratings of ten videos showcasing a variety of positive and negative cat-cat interactions; (2) describe the frequency of positive and negative cat-cat interactions in the home; and (3) assess factors (i.e. cat demographics, household environment) associated with obvious negative and positive cat-cat interactions in the home. Cat behaviour experts were also recruited to rate their interpretations of the ten videos.

We predicted that caregivers’ ratings of overt positive and negative inter-cat behaviours would be similar to expert ratings, while differences in ratings would be detected for more subtle interactions, such as resource guarding and nose touching. Factors predicted to be associated with a higher frequency of positive cat-cat interactions were cat-dyads which were related, younger, and have access to multiple resource areas (i.e. multiple litter-box and feeding areas) in the home. Conversely, factors predicted to be associated with a higher frequency of negative cat-cat interactions were cats that were older, unrelated, reported to have health or behavioural problems, and have limited provision of resources (i.e. one litter and feeding area) in the home.

## Materials and methods

### Ethical status

This study was reviewed by the University of California Davis Institutional Review Board to recruit human participants for research (IRB #1786341). Respondents provided consent to participate electronically prior to accessing the questionnaire, and participation was anonymous.

### Ethogram: Cat-cat interactions

An ethogram describing a range of positive and negative cat-cat interactions was developed (see Table S1; Supplementary material). The ethogram includes active whole-body behaviours, vocalisations, and specific movements of body parts, such as the eyes, ears, tail. The ethogram served as a tool during video selection to identify the valence (positive, negative) of cat behaviours displayed by cats in the videos.

### Video selection

Two authors (SK and MK) searched the website youtube.com to select videos showcasing a variety of interactions between two domestic cats. The authors initially searched for general terms such as ‘two cats’ and then over time narrowed down the searches to specific interactions such as ‘two cats playing indoors’. We did not pre-determine specific search terms, rather the two authors independently explored various search terms to find videos that met the following inclusion criteria: focuses on one interaction between two cats; both cats appear approximately one year of age or older (determined based on size and physical appearance); both cats’ bodies were fully visible during the interaction (with the exception of Video G where the end of the tail is out of frame); both cats appear physically healthy (no obvious physical health issues) and do not show anatomical anomalies (i.e. missing a leg or portion of the ear). Video exclusion criteria included: less than or more than two cats involved in the focal interaction; one or both cats not fully visible; one or both cats appear to be kitten age (< 1 year old); one or both cats appear to have physical health issues or anatomical anomalies; videos with distracting backgrounds (i.e. people, other animals, or moving vehicles in the background); videos with millions of views (in order to maintain novelty of the videos); and videos of poor quality (i.e. low-resolution, lots of camera movement). Additionally, we avoided selecting videos with visually identifiable purebred cats as they may have breed-specific traits. No further information was obtained from the cats in the videos.

A total of 38 videos met the inclusion criteria. The ethogram was used to identify the valence of behaviours for both cats in each video and to determine the overall valence (positive, negative) of the video. Then, each video was rated using a five-point Likert scale to assess the degree of cat-cat interaction (very subtle, moderately subtle, neither subtle nor obvious, moderately obvious, very obvious). Videos with unclear behaviours or interactions were removed, and video quality was re-checked to remove videos that lacked brightness, clarity, or sharpness. To ensure a range of cat-cat interactions, a video was selected to fit into each category of the five-point Likert scale for each valence group (positive, negative), resulting in ten total videos. Of the ten videos, five showcased affiliative cat-cat interactions (nose touch, head rub, playing, co-sleeping, and allo-grooming) and five showcased negative cat-cat interactions (resource guarding food, staring, resource guarding catnip, striking, and fighting), which ranged from very subtle to very obvious (for the video links, and a non-exhaustive list of behaviours identified from each interaction, please see Table 1).

**Table 1.** Ten videos showcasing a range of positive and negative cat-cat interactions categorised by valence (positive, negative) and degree of interaction (very subtle - very obvious). Edited versions of the original YouTube videos were used in the cat caregiver and cat behaviour expert questionnaires

Video	Valence	Degree of interaction	**Original video link**	Time stamp	Edited video link
A Nose touch	**+**	Very subtle	youtu.be/yEWy98KcCX8?si=p8zYblo--OUBcsfP	0:00–0:03	youtu.be/DCtg6QEGmfE
B Head rub	**+**	Moderately subtle	youtu.be/I1KnQau5RQw?si=oCt76Cg7AOv3xVTS	0:37–0:47	youtu.be/s8BJ0WaJ_J8
C Play	**+**	Neither subtle nor obvious	youtu.be/IETeb-Mw–28?si=2Ho0_D6whlwyHBkm	6:32–6:42	youtu.be/sW1E7jqv4X4
D Co-sleeping	**+**	Moderately obvious	youtu.be/i9Mf0-soonE?si=8viquugVHxSAsQ4F	0:21–0:25	youtu.be/4gjBqIyBI9M
E Allo-grooming	**+**	Very obvious	youtu.be/qI5KBGjmDIM?si=7ylzw_InDER0zD6v	0:15–0:25	youtu.be/qbuUzRt2PZc
F Guarding food	**-**	Very subtle	youtu.be/ttKdGMgf8y8?si=DEzHPqNTeuv-iXdp	5:01–5:10	youtu.be/K5EqiLs15sM
G Staring	**-**	Moderately subtle	youtu.be/6QNoDzojjkw?si=UH4a2wzkEadMACfZ	0:46–0.51	youtube.com/watch?v=BhCWSPUXnjs
H Guarding catnip	**-**	Neither subtle nor obvious	youtu.be/8ctFsY8255k?si=PXFwMrdOcjDk2elb	3:55–4:05	youtu.be/PA6PUe1b37s
I Striking	**-**	Moderately obvious	youtu.be/GuCRvLlgEVk?si=eT6PJKtCyohwcpyO	0:36–0:46	youtu.be/XCbkIyn5CfA
J Fighting	**-**	Very obvious	youtu.be/TtyFSsP90cw?si=JJrOX4zPeJgfembm	0:00–0:09	youtu.be/UsTr9Qln_mo

The final ten videos were edited (InShot Video Editor application, China): video quality was improved (i.e. adjust brightness), sound was removed to reduce distracting background noises, subtitles were added when a cat vocalisation occurred, duration of the video clip was reduced such that all videos were a maximum of 10-s long and only included one focal interaction. The edited videos were uploaded to YouTube as ‘unlisted’ so they could only be viewed using specific links, and then the links were embedded in an online questionnaire (Qualtrics Software Company, Provo, UT, USA).

### Cat caregiver questionnaire

The online questionnaire was comprised of five sections (n = 77 questions) which included: (1) inclusion criteria (n = 4); (2) cat-caregiver demographics (n = 9); (3) in-home resource provision and distribution (n = 4); (4) cat health, behaviour, and cat-cat relationship information (n = 20); and (5) rating the ten cat-cat interaction videos, and indicating the frequency of these interactions displayed in their own cats (n = 40). The full questionnaire is provided in the supplementary files of Khoddami *et al.* (2023). The current study focuses on section 5 of this questionnaire.

Briefly, sections 1–4 of the survey contained questions designed to collect participant demographic information such as age, gender, and country of residence; household data such as approximate household area, total number of adults, children, and dogs living in the home; participant ratings of self-perceived knowledge on inter-cat behaviour; and previous professional experience working with cats (if yes, the total number of years). Furthermore, we asked about participants’ perception of their cats’ overall relationship, their cats’ information such as breed and age, and current/previous health and behavioural problems.

Section 5 of the questionnaire included ten videos of cat-cat interactions (five negatively valenced and five positively valenced), as well as a snapshot of each video with the cat on the left labelled as cat 1, and the cat on the right as cat 2. The snapshot photographs were used to ask participants additional questions relating to the experience of each cat in the videos. Using a Likert scale (extremely negative, somewhat negative, neither positive nor negative, somewhat positive, extremely positive, not sure), participants were asked to rate: (1) the overall interaction between the two cats; (2) cat 1’s experience; and (3) cat 2’s experience. Lastly, for each video, participants were asked how frequently (never, rarely, sometimes, often, always, not sure) they see the behaviours in their own cats. Participants could re-watch the videos as many times as desired, and the order in which the videos appeared were randomised between participants.

Survey participation was voluntary, not compensated, and required informed consent from the participant. To be eligible for participation, individuals were required to be aged at least 18 years or older, currently residing in Canada or the USA, and the current primary caregiver of two companion cats who spend at least half of their time indoors. The questionnaire was provided in English and internet access was required for participation. Recruitment involved distributing an advertisement on social media sites, such as Facebook and Twitter, using the snowball sampling method (Biernacki & Waldorf 1981). The questionnaire was accessible from September 13 to September 17, 2021.

### Cat expert questionnaire

A cat behaviour expert online questionnaire (n = 51 questions; Qualtrics Software Company) was created which included the same questions as section 5 of the cat caregiver questionnaire (n = 40), with the exception of the question asking about seeing the behaviour in their own cats. In addition, a question asked to indicate their certifications (DVM DACVB, PhD, MS, CAAB, ACAAB) and an optional open-text box was provided to input comments after each video (n = 11). The expert ratings of the cat-cat interactions were used as ‘gold standard’ ratings to compare with the cat caregiver questionnaire ratings.

Survey participation was voluntary, anonymous, not compensated, and required informed consent. The authors invited five cat behaviour experts (those with advanced degrees in animal behaviour and have published on the topic of companion animal behaviour) residing in the US or Canada to participate. The experts were emailed an invitation to participate, which included a description of the research and a questionnaire link. Data collection occurred between June 11 and July 11, 2021.

### Statistical analysis

#### Cat caregiver questionnaire

Please see Khoddami *et al.* (2023) for details on statistical analyses and results for questionnaire sections 1–4. In brief, responses from 6,529 caregivers of two cats (n = 13,058 cats) who completed the full questionnaire were included in the analysis. During data cleaning, questions with the answer option ‘other’ and open text responses were evaluated to ensure accurate response allocation to reduce misclassification bias. Descriptive statistics were generated for each question using RStudio (Auckland, New Zealand) initially by country, then later combined due to similarity, and statistical tests were conducted using SAS Studio v3.7 (SAS Institute, Cary, NC, USA).

For questionnaire section 5, descriptive statistics were generated for each question. Four logistic regression models were then built to predict explanatory variables associated with participants’ ratings of how often they indicated seeing the cat-cat behaviours showcased in the videos, in their own cats. Given our predictions that cat caregivers would be better at identifying obvious positive and negative cat-cat interactions, the four outcome variables selected for the models were the two obvious negative interactions (striking, fighting) and two obvious positive interactions (allo-grooming, co-sleeping). For each outcome, the Likert-scale data were consolidated to create a binary outcome variable with ‘sometimes’, ‘often’ and ‘always’ combined into a ‘yes’ category, and ‘rarely’ and ‘never’ combined into a ‘no’ category; the ‘not sure’ category was not of interest and thus not included. Explanatory variables included in the model were those predicted to influence each outcome variable (n = 41), including cat characteristics, cats’ overall relationship, resource provision in the home, and health and behaviour variables. Explanatory variables with highly skewed response distributions were not included, and variables with a large number of response categories such as the ‘health problem’ variable were consolidated (i.e. health condition: yes, no). The variable ‘first encounter’ was excluded from all of the models due to misclassification bias. Due to the large number of explanatory variables, two-way analyses using a liberal *P*-value (*P* < 0.2) were used to guide inclusion of variables into each model. The models were built using a backwards model building strategy whereby variables with a *P* < 0.05 were retained and included predicted two-way interactions. Stepwise model building followed to ensure no significant variables or interactions were missed. Due to all explanatory variables being categorical, model fit was based on evaluation of the two-way interaction terms. *Post hoc* pair-wise comparisons with four or more pairs used a Tukey’s adjustment for multiple comparisons to reduce the potential for type I errors. Results are reported using odds ratios (OR), 95% CI’s and *P*-values.

#### Expert questionnaire

Descriptive statistics were generated for each question. The Kruskal Wallis test was then used to assess differences between the five behaviourists’ Likert scale ratings (overall scores, cat 1 scores, and cat 2 scores) for the ten videos.

#### Expert versus cat caregiver participant video ratings

The Wilcoxon two-sample test using Monte Carlo estimates for exact tests was used to assess differences between participant versus expert ratings (overall scores, cat 1 scores, cat 2 scores) for each of the ten videos. A continuity correction was used to correct for a non-symmetrical distribution about the median. The null hypothesis is that the two populations have the same distribution with the same median. If we reject the null, that means we have evidence that one distribution is shifted to the left or right of the other.

## Results

### Cat caregiver questionnaire sections 1–4: Descriptive results summary

Please refer to Khoddami *et al.* (2023) for complete reporting of the descriptive results for sections 1–4 of the questionnaire. In brief, of the total respondents (n = 6,529), the majority reside in the US (93.7%), identified as female (71.5%), lived in households with two adults (63.1%), had no dogs (76.4%) and no children (77.8%). Most participants were aged 30–39 or 40–49 years old (31.4, 24.4%, respectively), did not have professional working experience with cats (72.4%), and reported themselves as ‘very knowledgeable’ about cat behaviour (44.5%).

The majority of respondents’ cats (two per household, total of 13,058) were neutered males (49.8%) and spayed females (49.3%), and were 1 to 3 years old (30.6%), 4 to 6 years old (24.1%), 7 to 10 years old (24.0%), or 10 years or older (21.3%). Participants most frequently indicated that their cats were acquired at kitten age (73.8%), from a shelter (59.7%), were domestic breed (76.4%), had a tabby coat pattern (35.5%), and were strictly indoors-only (67.1%) or indoors with supervised outdoor access (23.5%).

Most respondents indicated that they provide their two cats with a single feeding area (59.1%), a single litter-box area (57.1%), multiple sleeping areas (83.4%), and multiple scratching posts (78.9%). In most households, at least one cat was reported to have ≥ 1 current or previously diagnosed health issue(s) (59.9%), with the most frequently reported issues being obesity (25.2%) and/or dental disease (23.4%). A large number of participants indicated at least one of their cats had ≥ 1 current or previous behavioural issue(s) (78.5%), including fears/phobias (45.7%), unwanted behaviours (45.2%), and/or destructive behaviours (40.6%). It is important to note the latter two may include normal cat behaviours that are viewed as problematic by caregivers.

### Cat caregiver questionnaire section 5: Cat video descriptive results summary

**Figure 1.**
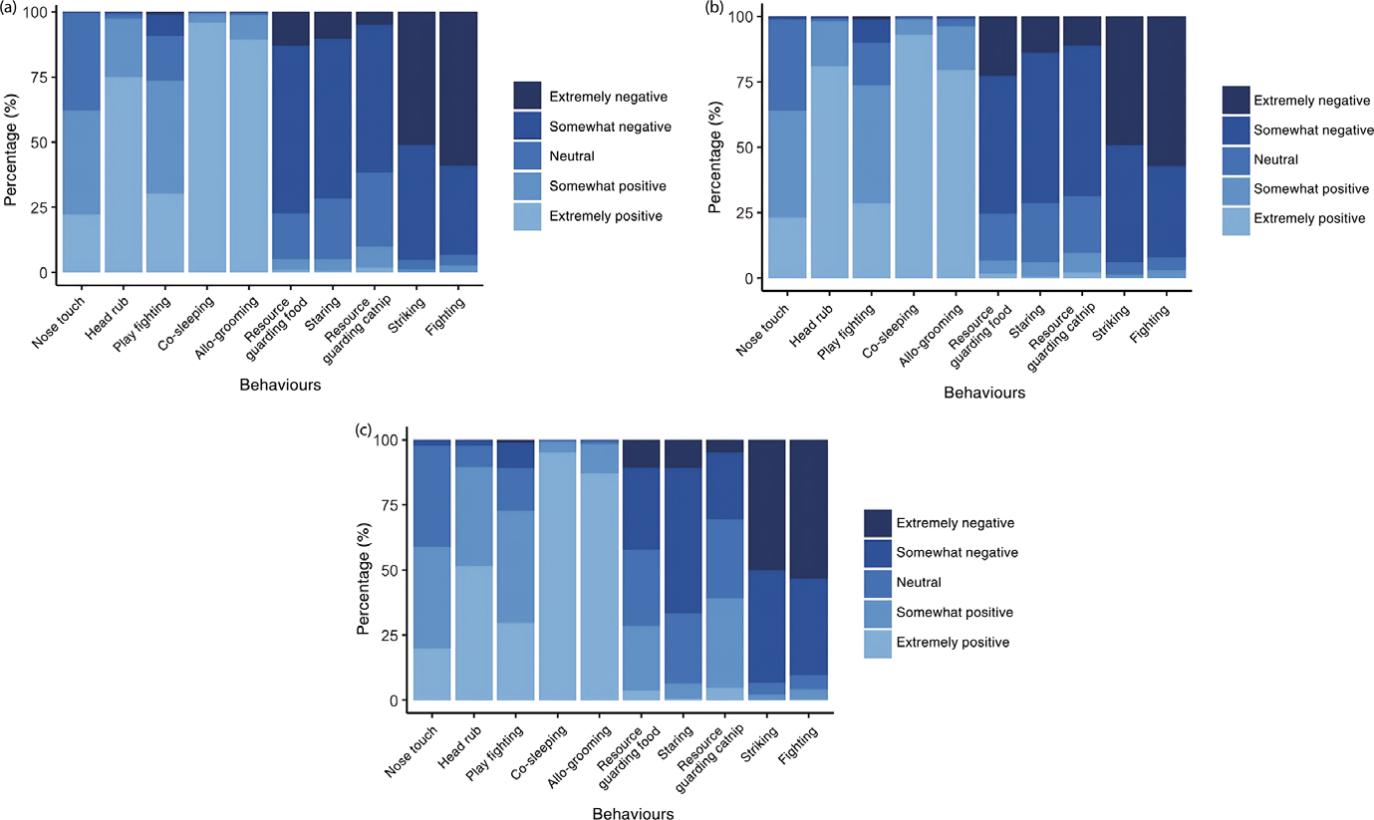
Graphical descriptive summary of participant cat caregivers’ (n = 6,529) Likert scale ratings for the ten cat-cat interaction videos for (a) the overall cat-cat interaction, (b) cat 1’s experience and (c) cat 2’s experience. The x-axis lists positive-valenced interactions first, followed by those that were negatively valenced, with interactions in each category arranged from very subtle to very obvious.

A graphical summary of participants’ video ratings are shown in Figure 1. To summarise, most respondents (> 50%) rated the overall interaction between the cats in the videos as extremely negative for striking and fighting, somewhat negative for videos showcasing guarding food, staring, and guarding catnip, somewhat positive for videos showing allo-grooming, co-sleeping, and head rub, and most frequently rated the nose touch as somewhat positive or neutral. Most respondents (> 50%) rated the experience of cat 1 in the videos as extremely negative for striking and fighting, somewhat negative for videos showcasing guarding food, staring, and guarding catnip, extremely positive for videos showing allo-grooming and co-sleeping, somewhat positive or extremely positive for play fighting, and most frequently rated nose touch as somewhat negative or neutral. Most respondents (> 50%) rated the experience of cat 2 in the videos as extremely negative for striking and fighting, staring as somewhat negative, allo-grooming and co-sleeping as extremely positive, and most frequently rated guarding catnip as somewhat positive or neutral, guarding food as somewhat negative, neutral, or somewhat positive, playing and head-rub as extremely positive or somewhat positive, and nose touch as somewhat positive or neutral.

**Figure 2.**
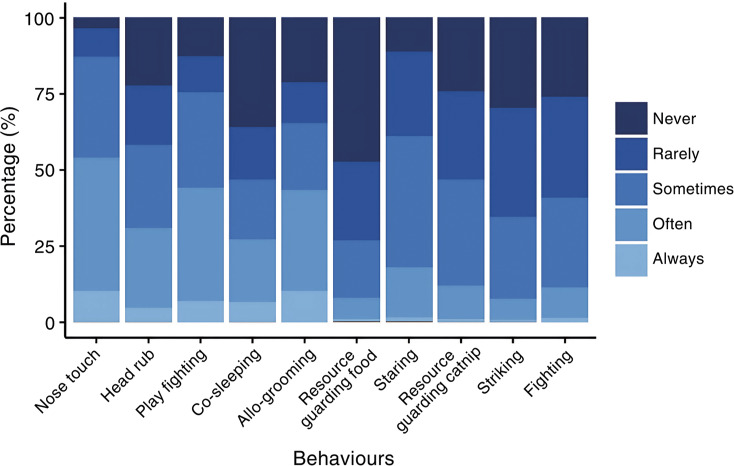
Graphical descriptive summary of participant cat caregivers’ (n = 6,529) Likert scale ratings of how frequently their two cats display the behaviours showcased in the ten cat-cat interaction videos. The x-axis lists positive-valenced interactions first, followed by those that were negatively valenced, with interactions in each category arranged from very subtle to very obvious.

A graphical summary of participants’ ratings of how often they see the video interactions in their own cats are shown in Figure 2. In summary, most participants (> 50%) reported their cats sometimes or rarely resource guard catnip. However, it is important to note that resource guarding might be expressed through other behaviours that require further exploration in cats (i.e. rapid ingestion or avoidance, explored in dogs [*Canis familiaris*] by Jacobs *et al.* 2018) which may not have been explicitly shown in the videos. Many participants reported that their cats stare at each other sometimes or rarely, while fighting and staring were reported to sometimes, rarely, or never be seen. Most respondents (> 50%) indicated their cats often or sometimes displayed nose touching, play fighting, and allo-grooming, while reporting their cats never or often co-sleep, and that head rubbing is seen often, sometimes, or never seen.

### Expert questionnaire

Five experts completed the full questionnaire and indicated the following certifications: Doctor of Philosophy and Certified Applied Animal Behaviourist (n = 3), Doctor of Veterinary Medicine and Diplomate of the American College of Veterinary Behaviourists (n = 1), Masters Degree and Associate Certified Applied Animal Behaviourist (n = 1). See Table 2 for a summary of experts’ video ratings for: (1) the overall cat-cat interaction (n = 10); (2) cat 1’s experience (n = 10); and (3) cat 2’s experience (n = 10).

**Table 2.** Cat expert (n = 5) Likert scale ratings for the ten cat-cat interaction videos for the overall cat-cat interaction, cat 1’s experience, and cat 2’s experience

Video	Overall interaction (%)		Cat 1’s experience (%)		Cat 2’s experience (%)		N
G Staring	Extremely positive	0	Extremely positive	0	Extremely positive	0	5
	Somewhat positive	0	Somewhat positive	0	Somewhat positive	0	
	Neither	0	Neither	0	Neither	0	
	Somewhat negative	80	Somewhat negative	80	Somewhat negative	80	
	Extremely negative	20	Extremely negative	20	Extremely negative	20	
C Play	Extremely positive	0	Extremely positive	0	Extremely positive	0	5
	Somewhat positive	100	Somewhat positive	100	Somewhat positive	80	
	Neither	0	Neither	0	Neither	20	
	Somewhat negative	0	Somewhat negative	0	Somewhat negative	0	
	Extremely negative	0	Extremely negative	0	Extremely negative	0	
B Head rub	Extremely positive	40	Extremely positive	80	Extremely positive	0	5
	Somewhat positive	60	Somewhat positive	20	Somewhat positive	80	
	Neither	0	Neither	0	Neither	20	
	Somewhat negative	0	Somewhat negative	0	Somewhat negative	0	
	Extremely negative	0	Extremely negative	0	Extremely negative	0	
A Nose touch	Extremely positive	0	Extremely positive	0	Extremely positive	20	5
	Somewhat positive	40	Somewhat positive	20	Somewhat positive	40	
	Neither	60	Neither	80	Neither	40	
	Somewhat negative	0	Somewhat negative	0	Somewhat negative	0	
	Extremely negative	0	Extremely negative	0	Extremely negative	0	
H Guard catnip	Extremely positive	0	Extremely positive	0	Extremely positive	0	5
	Somewhat positive	0	Somewhat positive	0	Somewhat positive	60	
	Neither	0	Neither	0	Neither	20	
	Somewhat negative	80	Somewhat negative	100	Somewhat negative	20	
	Extremely negative	0	Extremely negative	0	Extremely negative	0	
E Allo-groom	Extremely positive	60	Extremely positive	60	Extremely positive	60	5
	Somewhat positive	40	Somewhat positive	40	Somewhat positive	20	
	Neither	0	Neither	0	Neither	20	
	Somewhat negative	0	Somewhat negative	0	Somewhat negative	0	
	Extremely negative	0	Extremely negative	0	Extremely negative	0	
F Fight	Extremely positive	0	Extremely positive	0	Extremely positive	0	5
	Somewhat positive	0	Somewhat positive	0	Somewhat positive	0	
	Neither	0	Neither	0	Neither	0	
	Somewhat negative	20	Somewhat negative	20	Somewhat negative	20	
	Extremely negative	80	Extremely negative	80	Extremely negative	80	
D Co-sleep	Extremely positive	80	Extremely positive	80	Extremely positive	80	5
	Somewhat positive	0	Somewhat positive	0	Somewhat positive	0	
	Neither	20	Neither	20	Neither	20	
	Somewhat negative	0	Somewhat negative	0	Somewhat negative	0	
	Extremely negative	0	Extremely negative	0	Extremely negative	0	
I Strike	Extremely positive	0	Extremely positive	0	Extremely positive	0	5
	Somewhat positive	0	Somewhat positive	0	Somewhat positive	0	
	Neither	0	Neither	0	Neither	0	
	Somewhat negative	40	Somewhat negative	40	Somewhat negative	40	
	Extremely negative	60	Extremely negative	60	Extremely negative	60	
J Guard food	Extremely positive	0	Extremely positive	20	Extremely positive	0	5
	Somewhat positive	20	Somewhat positive	0	Somewhat positive	60	
	Neither	0	Neither	0	Neither	0	
	Somewhat negative	80	Somewhat negative	80	Somewhat negative	40	
	Extremely negative	0	Extremely negative	0	Extremely negative	0	

The Kruskal Wallis test results showed no significant differences between the five experts’ Likert scores for: overall cat-cat interactions (*P* = 0.54), cat 1’s experience (*P* = 0.77), and cat 2’s experience (*P* = 0.14).

### Video ratings: Expert versus participant scores

**Figure 3.**
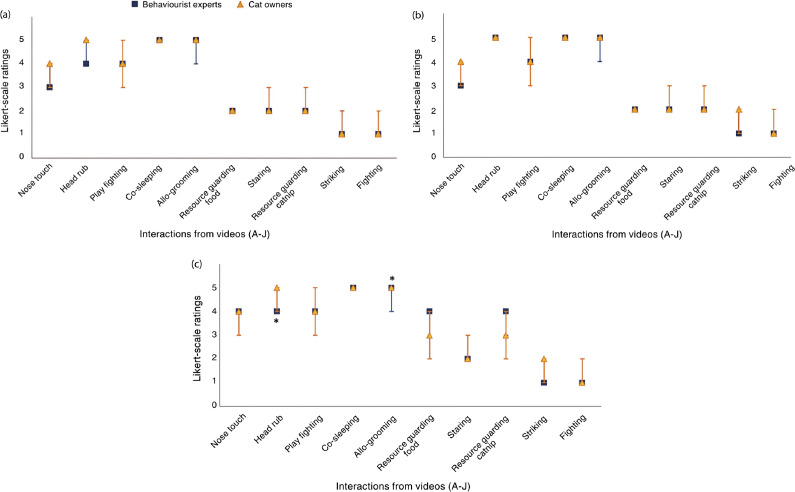
Cat caregivers’ (n = 6,529) and behaviour experts’ (n = 5) median (lower, upper quartiles) scores for the ten cat-cat interaction videos for (a) the overall interaction, (b) cat 1’s experience and (c) cat 2’s experience. Videos were scored on a Likert scale: 1 = extremely negative, 2 = somewhat negative, 3 = neither negative nor positive, 4 = somewhat positive, 5 = extremely positive. The Wilcoxon two-sample test using Monte Carlo estimates for exact tests and a continuity correction were used to assess differences between participant versus expert ratings. The x-axis lists positive-valenced interactions first, followed by those that were negatively valenced, with interactions in each category arranged from very subtle to very obvious. * Significance at *P* < 0.05.

Across all videos, no differences in rank-sums around the medians were detected between participants’ versus experts’ ratings of the overall cat-cat interactions and cat 1’s experience (*P* > 0.05; Figure 3). No differences were detected in rank-sums around the medians between participants’ and experts’ ratings for cat 2’s experience, except video B (head rub; *P* = 0.02) and video E (allo-grooming; *P* = 0.04).

### Questionnaire section 5: Logistic regression model

Factors associated with caregiver reports that their two-cats show fighting behaviour, as seen in video J, included: declaw status, cats’ age, litter-box provision, aggression towards animals, and cats’ perceived overall relationship (Table 3). Factors associated with caregiver reports that their two-cats show striking behaviour, as seen in video I, included: cats’ age, litter-box provision, aggression towards animals, cats’ perceived overall relationship, and the presence of dogs in the household (Table 4). Factors associated with caregiver reports that their two-cats show allo-grooming, as seen in video E, included: the amount of time cats have lived together, number of feeding areas in the home, cats’ age, cats’ sex, and perceived overall relationship (Table 5). Lastly, factors associated with caregiver reports that their two-cats show co-sleeping, as seen in video D, included: cats’ age, cats’ sex, the amount to time cats have lived together, number of feeding areas in the home, perceived overall relationship, outdoor access, and an interaction between animal aggression and cats’ relation was detected (Table 6). No other significant effects were detected.

**Table 3.** Multi-level logistic regression results showing factors significantly (*P* < 0.05) associated with two cats from the same household displaying fighting behaviour (as demonstrated in video J), based on caregiver reports (n = 6,529 participants). Odds ratios (OR) > 1 indicate increased odds, while OR < 1 indicate decreased odds compared to the referent. ORs, 95% CIs, and *P*-values reported. For explanatory variables with 4 or more response options, Tukey adjusted *P*-values and adjusted 95% confidence intervals are reported.

Explanatory variable	Category	OR (95% CI)	*P-*value
Animal aggression	No	Ref	
	Yes	1.6 (1.4–1.84)	<0.0001
Cats’ age	Both young	Ref	
	Adult & mature	1.33 (1.01–1.76)	0.041
	Both adult	1.48 (1.13–1.95)	0.0006
	Both mature	1.46 (1.17–1.83)	< 0.0001
	Young & adult	1.61 (1.2–2.18)	< 0.0001
	Young & mature	1.64 (1.22–2.2)	< 0.0001
Cats’ overall relationship	Neither positive nor negative	Ref	
	Negative	2.46 (2–3.03)	< 0.0001
	Positive	Ref	
	Neither positive nor negative	1.91 (1.65–2.22)	< 0.0001
	Negative	4.7 (3.96–5.59)	< 0.0001
Cats’ declaw status	None declawed	Ref	
	Both declawed	1.29 (1.03–1.62)	0.027
	One declawed	Ref	
	Both declawed	1.6 (1.15–2.21)	0.005
Litter-box areas	Multiple	Ref	
	Single	1.13 (1.01–1.25)	0.030

**Table 4.** Multi-level logistic regression results showing factors significantly (*P* < 0.05) associated with two cats from the same household displaying striking (as demonstrated in video I), based on caregiver reports (n = 6,529 participants). ORs, 95% CIs, and *P*-values reported. For explanatory variables with 4 or more response options, Tukey adjusted *P*-values and adjusted 95% confidence intervals are reported.

Explanatory variable	Category	OR (95% CI)	*P-*value
Animal aggression	No	Ref	
	Yes	1.5 (1.31–1.72)	<0.0001
Cats’ age	Both young	Ref	
	Adult & mature	1.42 (1.06–1.89)	0.0077
	Both adult	1.39 (1.04–1.85)	0.0142
	Both mature	1.55 (1.23–1.96)	< 0.0001
	Young & adult	1.46 (1.07–2)	0.0074
	Young & mature	1.49 (1.1–2.02)	0.0026
Cats’ overall relationship	Neither positive nor negative	Ref	
	Negative	2.13 (1.74–2.59)	< 0.0001
	Positive	Ref	
	Neither positive nor negative	1.96 (1.69–2.28)	< 0.0001
	Negative	4.17 (3.54–4.91)	< 0.0001
Dogs	Yes	Ref	
	No	1.27 (1.12–1.44)	0.0003
Litter-box areas	Multiple	Ref	
	Single	1.13 (1.01–1.26)	0.0289

**Table 5.** Multi-level logistic regression results showing factors significantly (*P* < 0.05) associated with two cats from the same household displaying allo-grooming behaviour (as demonstrated in video E) based on caregiver reports (n = 6,529 participants). ORs, 95% CIs, and *P*-values reported. For explanatory variables with 4 or more response options, Tukey adjusted *P*-values and adjusted 95% confidence intervals are reported.

Explanatory variable	Category	OR (95% CI)	*P-*value
Cats’ sex	Both spayed females	Ref	
	Neutered male & spayed female	1.6 (1.28–1.99)	< 0.0001
	Both neutered males	2.91 (2.22–3.81)	< 0.0001
	Neutered male & spayed female	Ref	
	Both neutered males	1.82 (1.43–2.33)	< 0.0001
Cats’ relation	Not related	Ref	
	Other	1.64 (1.05–2.56)	0.024
	Siblings	2.04 (1.63–2.56)	< 0.0001
Time together (years)	1–3 years	Ref	
	10+ years	4.74 (2.88–7.81)	< 0.0001
	4–6 years	2.37 (1.63–2.56)	< 0.0001
	7–9 years	5.07 (1.03–1.62)	< 0.0001
	Less than 1 year	Ref	
	1–3 years	1.95 (1.37–2.79)	< 0.0001
	10+ years	9.26 (5.09–16.85)	< 0.0001
	4–6 years	4.64 (2.86–7.51)	< 0.0001
	7–9 years	9.9 (5.48–17.86)	< 0.0001
	4–6 years	Ref	
	10+ years	2 (1.28–3.12)	0.0002
	7–9 years	2.13 (1.38–3.31)	< 0.0001
Animal aggression	Yes	Ref	
	No	1.53 (1.29–1.8)	< 0.0001
Cats’ age	Both mature	Ref	
	Adult & mature	2.12 (1.37–3.29)	< 0.0001
	Both adult	2.59 (1.62–4.15)	< 0.0001
	Both young	9.4 (5.56–15.89)	< 0.0001
	Young & adult	5.97 (3.51–10.17)	< 0.0001
	Young & mature	3.75 (2.24–6.29)	< 0.0001
	Adult & mature	Ref	
	Both young	4.43 (2.79–7.05)	< 0.0001
	Young & adult	2.82 (1.76–4.5)	< 0.0001
	Young & mature	1.77 (1.13–2.78)	0.004
	Both adult	Ref	
	Both young	3.62 (2.24–5.86)	< 0.0001
	Young & adult	2.3 (1.39–3.82)	< 0.0001
	Young & adult	Ref	
	Both young	1.57 (1.06–2.34)	0.014
	Young & mature	Ref	
	Both young	2.5 (1.71–3.66)	< 0.0001
	Young & adult	1.59 (1.07–2.36)	0.010
Cats’ overall relationship	Negative	Ref	
	Neither positive nor negative	2.38 (1.07–2.36)	< 0.0001
	positive	18.05 (14.43–22.58)	< 0.0001
	Neither positive nor negative	Ref	
	positive	7.58 (6.39–9.00)	< 0.0001
Feeding areas	Multiple	Ref	
	Single	1.41 (1.61–1.23)	< 0.0001
Household area	More than 1,500 sq ft	Ref	
	500–1,000 sq ft	1.28 (1.08–1.52)	0.036

**Table 6.** Multi-level logistic regression results showing factors significantly (*P* < 0.05) associated with two cats from the same household displaying co-sleeping behaviour (as demonstrated in video D), based on caregiver reports (n = 6,529 participants). ORs, 95% CIs, and *P*-values reported. For explanatory variables with 4 or more response options, Tukey adjusted *P*-values and adjusted 95% confidence intervals are reported.

Explanatory variable	Category	OR (95% CI)	*P-*value
Cats’ age	Both mature	Ref	
	Both adult	1.76 (1.11–2.8)	0.0068
	Both young	6.35 (3.74–10.78)	< 0.0001
	Young & adult	3.68 (2.14–6.33)	< 0.0001
	Young & mature	2.39 (1.38–4.12)	< 0.0001
	Adult & mature	Ref	
	Both young	4.34 (2.75–6.86)	< 0.0001
	Young & adult	2.52 (1.57–4.03)	< 0.0001
	Young & mature	1.63 (1.02–2.63)	0.038
	Both adult	Ref	
	Both young	3.6 (2.28–5.7)	< 0.0001
	Young & adult	2.09 (1.28–3.41)	0.0003
	Young & adult	Ref	
	Both young	1.73 (1.23–2.42)	< 0.0001
	Young & mature	Ref	
	Both young	2.66 (1.88–3.75)	< 0.0001
	Young & adult	1.54 (1.06–2.24)	0.013
Cats’ sex	Both spayed females	Ref	
	Neutered male & spayed female	1.34 (1.08–1.65)	0.0014
	Both neutered males	2.05 (1.62–2.59)	<0.0001
	Neutered male & spayed female	Ref	
	Both neutered males	1.53 (1.25–1.88)	< 0.0001
Time together (years)	1–3 years	Ref	
	10+ years	3.58 (2.14–6.01)	< 0.0001
	4–6 years	2.22 (1.52–3.24)	< 0.0001
	7–9 years	3.37 (2.03–5.59)	< 0.0001
	Less than 1 year	Ref	
	1–3 years	1.78 (1.27–2.51)	< 0.0001
	10+ years	6.39 (3.48–11.73)	< 0.0001
	4–6 years	3.95 (2.42–6.45)	< 0.0001
	7–9 years	6 (3.3–10.91)	< 0.0001
	4–6 years	Ref	
	10+ years	1.62 (1.03–2.53)	0.028
Feeding areas	Multiple	Ref	
	Single	1.28 (1.44 –1.13)	<0.0001
Cats’ overall relationship	Negative	Ref	
	Neither positive nor negative	2.56 (1.72–3.82)	<0.0001
	Positive	21.52 (15.2–30.47)	< 0.0001
	Neither positive nor negative	Ref	
	Positive	8.41 (6.76–10.45)	< 0.0001
Cats’ outdoor access	Both outdoor	Ref	
	Both indoor	1.28 (1.12–1.46)	0.0002
	One indoor, One outdoor	Ref	
	Both indoor	1.4 (1.11–1.76)	0.0042
Animal aggression × cats’ relation	No × Not related	Ref	
	No × Other	2.1 (1.27–3.49)	0.0004
	No × Siblings	1.92 (1.51–2.45)	< 0.0001
	Yes × Siblings	1.97 (3.31–1.17)	0.0026
	Yes × Not related	Ref	
	No × Not related	1.83 (1.38–2.43)	< 0.0001
	No × Other	3.86 (2.21–6.74)	< 0.0001
	No × Siblings	3.53 (2.53–4.93)	< 0.0001
	Yes × Siblings	3.61 (6.36–2.05)	< 0.0001

## Discussion

### Caregiver knowledge of cat-cat interactions

Overall, cat caregivers rated the valence of videos showcasing cat-cat interactions similarly to experts, with the exception of two affiliative interactions focused on cat 2’s experience: head rubbing and allo-grooming. Although we predicted differences in ratings for more subtle interactions such as head rubbing, we did not expect differences for obvious positive interactions such as allo-grooming. Research by Van Belle and colleagues (2023) using participatory videos from 42 two-cat households found that one in three caregivers under-reported cat-cat interactions. This research revealed head rubbing and allo-grooming to be the interactions most commonly overlooked by caregivers, which was identified through expert behavioural observations. The authors suggest these behaviours may be relatively subtle; head rubbing occurs in short bouts (Mertens 1991) and allo-grooming takes place in the absence of vocalisations (van den Bos 1998), and thus may be missed by caregivers (Van Belle *et al.* 2023). In addition, these interactions may be overlooked due to a lack of familiarity since they may not occur frequently in household cats (Crowell-Davis *et al.* 2004). Allo-rubbing is suggested to commonly occur in feral cats after periods of separation when conspecifics reunite (Crowell-Davis *et al.* 2004; Behnke *et al.* 2021). It is possible that our participants see these behaviours less commonly in their cats since our inclusion criteria specified their two cats must spend at least 50% of their time indoors. Therefore, participants’ cats may have reduced frequencies of cat-cat separation given the time they spend indoors together, compared to feral cats.

Although the majority of participants rated cat 2’s experience during allo-grooming as extremely positive, there was a variation in expert scores ranging from extremely positive, somewhat positive, to neither positive nor negative. In the context of the allo-grooming video, cat 2 groomed cat 1 and engaged in active licking behaviour. In general, allo-grooming is a social affiliative behaviour that is thought to strengthen the bond between cats (Curtis *et al.* 2003; Bradshaw 2016) and has been negatively associated with inter-cat aggression in multi-cat households (Elzerman *et al.* 2020). However, in some contexts, allo-grooming may be followed by an aggressive interaction. In a study by van den Bos (1998) involving a colony of neutered cats (n = 83), agonistic behaviour occurred in 35% of allo-grooming interactions, with the grooming cat often displaying some offensive behaviour (e.g. chasing, growling). Overall, more research is needed to elucidate the function of allo-grooming and rubbing, as well as the contexts in which these occur between indoor companion cats.

Contradictory to our predictions, most respondent ratings of negative cat-cat interactions did not differ from expert ratings. Research into Italian cat caregivers showed respondents had more difficulty identifying subtle negative cat responses, such as freezing and mydriasis, compared to more obvious negative responses such as excessive vocalisations and ears back (Mariti *et al.* 2017). Similarly, research suggests dog caregivers are better at identifying obvious negative dog responses that involve gross body movements such as trembling and whining, compared to more subtle signals, such as yawning and nose licking (Mariti *et al.* 2012). Given our results were not in line with current research, it is possible our participants were paying closer attention to the agonistic interactions, a psychological phenomenon known as negativity bias (Rozin & Royzman 2001), or perhaps those choosing to participate were more interested and knowledgeable regarding cat behaviour. Although this bias may exist, we had a large sample size which increases the representativeness of our results. Moreover, we had a small sample of cat behaviour experts, and future research of this type should include a larger expert sample to improve the precision of expert ratings.

### Cat-specific factors

Cat caregivers in this study appeared to have a good understanding of their cats’ relationship, as evidenced by their ratings of their cats’ overall relationship correlating with observed behaviours. For example, caregivers who perceived their cats as having a positive relationship were more likely to report seeing allo-grooming and co-sleeping behaviours, while those reporting a negative relationship were more likely to report fighting and striking behaviours. Supporting this, cats reported to show aggression towards other animals were less likely to engage in allo-grooming and more likely to engage in fighting and striking behaviours. Concerningly, 23% of study participants reported that at least one of their cats displays aggression towards other animals (see Table 6 in Khoddami *et al.* 2023); this is especially alarming if the aggression is directed at another household pet, which may negatively affect pet welfare and the human-animal bond (Salman *et al.* 1998, 2000).

Participants with older cat dyads, or dyads with substantial age gaps, were more likely to report seeing fighting and striking behaviours, and less likely to report allo-grooming and co-sleeping. This is consistent with our previous findings suggesting that caregivers are more likely to report a negative relationship for cat dyads in these age groups (Khoddami *et al.* 2023). It has also been suggested that younger cats may be less prone to aggression due to increased sociability and adaptability (Landsberg *et al.* 2012). Maturation, typically occurring between 2 to 4 years of age, may potentially increase social tensions and is anecdotally considered a risk factor for inter-cat aggression (Crowell-Davis & Stelow 2023); although there is currently no scientific evidence to support this claim. In addition, changes in social dynamics may occur as cats age; for example, health issues in senior cats may trigger aggressive encounters due to pain (Landsberg *et al.* 2012).

Participants with two neutered males, or a neutered male and spayed female pair, were more likely to report allo-grooming and co-sleeping between their cats compared to those with two spayed female cats. This corresponds with other findings in the current dataset (Khoddami *et al.* 2023), which revealed that caregivers perceived their cats as having a more positive relationship if the cats were two neutered males or a neutered male and a spayed female pair, compared to caregivers with two spayed female cats. While some research suggests male dyads may get along better than other sex combinations (Barry & Crowell-Davis 1999; Wassink-van der Schot *et al.* 2016), others suggest males are more likely to act as aggressors (Lindell *et al.* 1997). Since a cat’s sex may impact interactions with other cats, it is important for caregivers to consider both social and environmental factors (i.e. cat age, resource allocation in the home), as well as cat-cat introduction methods when acquiring a second cat. Currently, there is a paucity of scientific information to inform cat-cat introductions, and research incorporating behavioural observations of cat-cat dyads in the home is needed to better understand how various factors impact the cat-cat relationship.

Caregivers of related cats (i.e. siblings) were more likely to report seeing allo-grooming between their cats than those with unrelated cats. Additionally, cats that lived together for longer periods, such as over ten years, were more likely to engage in allo-grooming and co-sleeping than those together for shorter periods. Past research underscores the importance of early socialisation, as well as familiarity and relatedness on cats’ social behaviours (Finka 2022; Curtis *et al.* 2003). For example, relation and familiarity are significant factors for colony cats engaging in allo-grooming and staying in close proximity (Bradshaw & Hall 1999; Curtis *et al.* 2003). Similarly, other results from this dataset (Khoddami *et al.* 2023) suggest related cats are more likely to have a positive caregiver-perceived relationship compared to unrelated cats. Related cats likely develop a bond during the early socialisation period (~2–9 weeks), which may aid in promoting a positive relationship (Bradshaw & Hall 1999; Finka 2022). Thus, it is important for cat caregivers and cat rescue organisations to consider adopting/homing cats that are related or have an established bond wherever feasible.

In households where one or both cats were declawed, there was increased likelihood of reported fighting. This is not surprising, given that declawing has been associated with pain and behavioural problems, including increased aggression and risk of biting (Martell-Moran *et al.* 2018). The American Veterinary Medical Association (AVMA) discourages, and the Canadian Veterinary Medical Association (CVMA) strongly opposes, non-medically necessary declawing of cats due to associated health and welfare concerns (CVMA 2022; AVMA 2020). Cat caregivers report that household damage, such as scratching furniture, is the main reason they choose to declaw their cats (Yeon *et al.* 2001). Thus, educating caregivers on providing appropriate scratching items and the use of positive reinforcement training is crucial for preventing and managing unwanted scratching (Cisneros *et al.* 2022).

Cat dyads that were indoors-only had an increased likelihood of caregiver reported co-sleeping compared to cats with outdoor access. It has been suggested that outdoor cats may bring home unfamiliar scents, triggering aggression between household cats (Crowell-Davis & Stelow 2023). An alternative explanation could be that cats with outdoor access may spend less time together than indoor cats, which reduces the prevalence and/or duration of contact. However, more research is needed to understand the impact of different types of outdoor access (i.e. supervised versus unsupervised) on the cat-cat relationship.

### Resource-related factors

Interestingly, caregivers were more likely to report allo-grooming in cats living in households with an area of 500–1,000 square feet compared to those with over 1,500 square feet. Although larger areas for group-housed cats are shown to promote play and general activity (Loberg & Lundmark 2016), utilising vertical spaces, such as by adding shelves, is suggested to reduce agonistic interactions (Desforges *et al.* 2016). It is possible thus that in households where cats’ minimum space requirements are met, the availability of resources in the area may be more valuable than the size of the space itself.

Participants that provided a single cat feeding area were more likely to report allo-grooming and co-sleeping between their cats, compared to those with multiple feeding areas. While the temporal direction of this association remains uncertain, it is unlikely that a single feeding area causes a positive relationship, as adult cats are solitary hunters and typically do not share food (Bradshaw 2016; Delgado & Dantas 2020). It is plausible that caregivers feed cats together when they perceive their relationship positively. Given the domestic cat’s innate hunting motivation (Tschanz *et al.* 2011; Cecchetti *et al.* 2021), it is recommended that household cats are fed individually to prevent competition and agonism (Sadek *et al.* 2018; Houpt 2023). In multi-cat households, having multiple resources dispersed throughout the home is advised, such as the total number of food bowls equaling the number of cats plus one (Rochlitz 2007; Damasceno & Genaro 2014). However, studies indicate that many cat caregivers do not adhere to this recommendation for food bowls (31/55; 56.4%; Alho *et al.* 2016; 1,560/2,492; 62.6%; Elzerman *et al.* 2020), consistent with our findings (3,859/6,529; 59.1%; Khoddami *et al.* 2023).

Caregivers providing cats with a single litter-box were more likely to report their cats’ display fighting and striking compared to caregivers providing multiple litter-box areas. This contrasts with other findings suggesting that multiple litter-box areas are associated with a negatively perceived cat-cat relationship (Khoddami *et al.* 2023). However, the causation and temporality of this relationship remains undetermined, highlighting the need for longitudinal studies. Veterinary behaviourists recommend providing multiple, dispersed litter-boxes (Crowell-Davis & Stelow 2023) as cats prefer time-sharing resources rather than sharing them at a given point in time (Bernstein & Strack 1996; Loberg & Lundmark 2016). Despite this, many multi-cat households provide a single litter-box area (Barcelos *et al.* 2018; Grigg & Kogan 2019; Elzerman *et al.* 2020; Khoddami *et al.* 2023), underscoring the need for further research into the impact of resource provision on cat-cat relationships to establish evidence-based recommendations.

Participants with no dogs in the household were more likely to report striking in their cats compared to those with at least one dog. Cats and dogs are found to form amicable relationships (Thomson *et al.* 2018), and it is possible another household pet may help alleviate tension between cat dyads. However, there is a lack of research exploring the way in which this dynamic impacts the cat-cat relationship and further research is needed.

### Study limitations

First, this was a cross-sectional survey of caregiver reports, which limits the ability to establish cause-and-effect or temporality of relationships found. However, this methodology allowed us to reach a large sample of cat caregivers, something that is not possible with other methodologies, such as cat behaviour observation. The questionnaire was advertised on social media sites and snowball sampling allowed the advertisement to be shared by other sources (i.e. news article by Gizmodo). However, those not using social media or other web-based electronic means to access information may have been excluded. In addition, social desirability bias and recall bias may skew results, and thus it is possible that some participant responses may not reflect true household and cat information. Further, removing sound from videos and adding subtitles for cat vocalisations may introduce bias. Adding subtitles does not allow the participant to decipher the type of vocalisation and thus may impact interpretation of the cat-cat interactions. However, we chose to do this to remove bias from distracting background noise and improve accessibility of the videos for participants who may be hard of hearing or may not be able to play the videos with sound.

### Animal welfare implications

Our results highlight that the welfare of cats in two-cat households may be influenced by many factors, including cat dyad age and sex, and cat management such as resource allocation and outdoor access. These results may be used to inform cat adoption strategies, in-home management strategies and, overall, promote a positive cat-cat relationship in the home.

In addition, the current findings increase our understanding of cat caregivers’ interpretations of cat-cat interactions, and suggest interpretations are similar to experts for many negative and positive behavioural interactions. Although more research is needed to replicate this result, caregiver reports of cat behaviour may be valuable in many contexts, including at the veterinary clinic for understanding cat behaviour and the cat relationship at home.

## Conclusion

In conclusion, the current study explored factors associated with the frequency of positive and negative cat-cat interactions in two-cat households and assessed cat caregivers’ ratings of cat-cat interactions via videos. Although our results suggest that caregivers’ ratings of cat-cat interactions displayed in the ten videos were similar to experts’ ratings, caregivers perceived allo-grooming and head rubbing more positively than experts. Additionally, participant cat caregivers reported that their cats engage more frequently in positive interactions such as nose touching, play fighting, and allo-grooming, while co-sleeping and head rubbing were less common. Caregiver reports of negative interactions suggest staring and resource guarding catnip were more frequent than striking, fighting, and resource guarding food.

Various factors were identified as significantly associated with overt affiliative (allo-grooming and co-sleeping) and agonistic (striking and fighting) interactions. Notably, positive cat-cat interactions were more likely to be reported in cats that were related, living together for a long period, neutered male pairs, indoor-only, and had a single feeding area. Conversely, negative interactions were more likely reported for pairs of older cats, dyads with a large age gap, dyads with at least one declawed cat, a single litter-box area in the home, and dyads with a least one cat that shows animal-based aggression. The findings also suggest that cat caregivers have a good understanding of their cats’ relationships, as evidenced by the consistency between their perceptions and reported interactions. These findings contribute to a better understanding of the dynamics between cat dyads in households. However, to better understand the impact of various factors on cat-cat interactions, longitudinal cohort studies with two-cat households are needed.

## Supporting information

Khoddami et al. supplementary material Khoddami et al. supplementary material

## Supplementary material

The supplementary material for this article can be found at http://doi.org/10.1017/awf.2024.58.
