# Supplementary material for: US and Canadian cat caregiver’s ratings of cat-cat interactions: A video-based survey
Source: Anim Welf. 2024 Dec 20;33:e64. doi: 10.1017/awf.2024.58 (PMC11704566; doi:10.1017/awf.2024.58)
Supplement: Khoddami et al. supplementary material [file S0962728624000587sup001.pdf]

Supplementary material

US and Canadian cat caregiver's ratings of cat-cat interactions: A video-based survey

Sherry Khoddami[1,2], Makayla C Kiser[2], Carly M Moodyhttps://orcid.org/0000-0003-3444-5932[2]

[1] Faculty of Land and Food Systems, University of British Columbia, Vancouver, BC, Canada

[2] Department of Animal Science, University of California, Davis, CA, USA

Author for correspondence: Carly M Moody, email: cmoody@ucdavis.edu

Table S1. Ethogram of common positive and negative cat-cat interactions and behaviours that were used to select videos for inclusion into the questionnaire.

Type	Behaviour	Valence	Definition	References
Interaction	Fight	-	Two cats engage in physical contact, often grappling with one another, scratching and biting as they turn over; may include negative vocalisations	Stanton *et al.* (2015)
	Bite	-	Cat snaps teeth at or succeeds in nipping another cat	Stanton *et al.* (2015)
	Striking	-	Cat uses front paw to strike (contact made) or strike at (no contact made) another cat	Mellen (1993)
	Displace	-	Cat displaces another cat, causing an avoidance behaviour from them	Stanton *et al.* (2015)
	Stare	-	One cat fixedly looks at another without being easily distracted by other activity around	Mellen (1993)
	Face-off	-	Cats are face-to-face and staring directly at each other, within less than two body lengths away from each other	Mellen (1993)

	Retreat	-	Cat backs away from another cat while continually watching another cat	Stanton *et al.* (2015)
	Co-sleeping	+	Two cats sleeping in close contact curled up, intertwined, or on top of the other	Ley (2015)
	Allo-grooming	+	One cat licks the face, head, or body of another cat	Mellen (1993); Wolfe (2001)
	Head rub	+	Cat rubs forehead region against another cat or against an inanimate object	Mellen (1993)
	Nose touch	+	One or both cats approach and mutually touch or sniff each other's noses. This is often performed while standing and facing one another	Crowell-Davis *et al.* (2004)
	Social play	+	Cats mutually participate in chasing, pouncing, or wrestling without hissing, growling, or other aggressive vocalisations	Wolfe (2001)
Body posture	Crouch	-	Cat is alert and positioned with body close to the ground, legs bent close to the body	Stanton *et al.* (2015)
	Displacement behaviours	-	Cat displays self-maintenance behaviour out of context, including grooming, scratching, lip-licking or yawning	Rigterink (2023)
	Piloerection	-	Cat raises hairs on the back of their neck, shoulder, back or tail to give a fluffed-out appearance. More extreme situations have a greater proportion of erect hairs	Fernandez De Molina & Hunsperger (1959)
	Rolling onto back	+	Cat lies on back, belly up, all four limbs held in a semi-vertical position, tail moves back and forth, and body rolls side to side. Negative vocalisations such as hissing and growling are absent	West (1974)
Ear position	Flattened	-	Ears flattened against skull	Ley (2015)
	Forward	+	Ears straight up, facing forward	Ley (2015)
	Rotated back	Uncertain	Ears straight up, facing back but not flattened	Ley (2015)
Tail movement	Curled/tucked	-	Tail wrapped around the body and tucked under hind legs.	Ley (2015); Brown & Bradshaw (2014)
	Quivering	-	Tail held at 45–90° and quivers during spraying of urine	Brown & Bradshaw (2014)
	Swishing	-	Tail swishes violently from side to side	Brown & Bradshaw (2014); Cameron-Beaumont (1997)

	Vertical	+	Tail is held up vertically	Brown & Bradshaw (2014)
Vocalization	Growling	-	Low pitched, tense in the throat, rumbling sound. May sometimes begin with a drawn out [m] sound	Moelk (1944)
	Hissing	-	Repeated and raucous hissing sound	Brown *et al.* (1969); Fernandez De Molina & Hunsperger (1959)
	Snarl	-	Heavy, rapid intake of breath halting suddenly with an [o] sound	Moelk (1944)
	Spitting	-	Forced, rapid expiration from a slightly opened mouth [fft], coming to a sudden halt	Magoun *et al.* (1937); Moelk (1944)
	Wailing	-	Long and drawn out [wa-ou], initially ending in an [a] sound and becomes intensified towards the [ou]	Moelk (1944)

References

Brown JL, Hunsperger RW and Rosvold HE 1969 Defence, attack, and flight elicited by electrical stimulation of the hypothalamus of the cat. *Experimental Brain Research* **8**: 113–129. https://doi.org/10.1007/BF00234534

Brown SL and Bradshaw JWS 2014 Communication in the domestic cat: within- and between-species. In: Turner DC and Bateson P (eds) *The Domestic Cat: The Biology of its Behaviour* pp 37–63. Cambridge University Press: Cambridge, UK. http://eprints.soton.ac.uk/id/eprint/463206

Cameron-Beaumont CL 1997 *Visual and tactile communication in the Domestic cat (*Felis silvestris catus*) and undomesticated small felids*. PhD dissertation, University of Southampton, UK

Crowell-Davis SL, Curtis TM and Knowles RJ 2004 Social organization in the cat: a modern understanding. *Journal of Feline Medicine and Surgery* **6**: 19–28. https://doi.org/10.1016/j.jfms.2003.09.013

Fernandez De Molina AF and Hunsperger RW 1959 Central representation of affective reactions in forebrain and brain stem: electrical stimulation of amygdala, stria terminalis, and adjacent structures. *The Journal of Physiology* **145**: 251–265. https://doi.org/10.1113/jphysiol.1959.sp006140

Ley JM 2015 Feline communication. In Rodan I and Heath S (eds) *Feline Behavioral Health and Welfare* pp 34–40. Elsevier: Amsterdam, The Netherlands.

Magoun HW, Atlas D, Ingersoll EH and Ranson SW 1937 Associated facial, vocal and respiratory components of emotional expression: an experimental study. *Journal of Neurology and Psychopathology* **17**: 241–255. https://doi.org/10.1136/jnnp.s1-17.67.241

Mellen JD 1993 A Comparative analysis of scent-marking, social and reproductive behavior in 20 species of small cats (*Felis*). *American Zoologist* **33**: 151–166. https://doi.org/10.1093/icb/33.2.151

Moelk M 1944 Vocalizing in the house-cat; a phonetic and functional study. *The American Journal of Psychology* **57**: 184–205. https://doi.org/10.2307/1416947

Rigterink A 2023 Fear, anxiety, stress behaviors in cats. In: Crowell-Davis S and Stelow E (eds) *Clinical Handbook of Feline Behavior Medicine* pp 129–141. John Wiley & Sons: New Jersey, USA. https://doi.org/10.1002/9781119653271.ch8

Stanton LA, Sullivan MS and Fazio JM 2015 A standardized ethogram for the felidae: A tool for behavioral researchers. *Applied Animal Behaviour Science* **173**: 3–16. https://doi.org/10.1016/j.applanim.2015.04.001

West M 1974 Social play in the domestic cat. *American Zoologist* **14**: 427–436. https://doi.org/10.1093/icb/14.1.427

Wolfe RC 2001 *The social organization of the free-ranging domestic cat (*Felis catus*).* PhD dissertation, University of Georgia, Athens, USA. https://www.proquest.com/openview/437149e2c461a9a2a88b654c42ff9db1/1?pq-origsite=gscholar&cbl=18750&diss=y
